# The Roles of *GmERF135* in Improving Salt Tolerance and Decreasing ABA Sensitivity in Soybean

**DOI:** 10.3389/fpls.2019.00940

**Published:** 2019-07-23

**Authors:** Meng-Jie Zhao, Li-Juan Yin, Jian Ma, Jia-Cheng Zheng, Yan-Xia Wang, Jin-Hao Lan, Jin-Dong Fu, Ming Chen, Zhao-Shi Xu, You-Zhi Ma

**Affiliations:** ^1^Institute of Crop Science, Chinese Academy of Agricultural Sciences (CAAS)/National Key Facility for Crop Gene Resources and Genetic Improvement, Key Laboratory of Biology and Genetic Improvement of Triticeae Crops, Ministry of Agriculture, Beijing, China; ^2^Department of Agronomy, Jilin Agricultural University, Changchun, China; ^3^College of Agriculture, Anhui University of Science and Technology, Fengyang County, China; ^4^Hebei Academy of Agriculture and Forestry Sciences, Research Center of Wheat Engineering Technology of Hebei, Shijiazhuang, China; ^5^College of Agronomy and Plant Protection, Qingdao Agricultural University, Qingdao, China

**Keywords:** ABA, ethylene-responsive factor, hypocotyl elongation, root growth, response mechanism, salt tolerance, soybean

## Abstract

Abscisic acid (ABA) mediates various abiotic stress responses, and ethylene responsive factors (ERFs) play vital role in resisting stresses, but the interaction of these molecular mechanisms remains elusive. In this study, we identified an ABA-induced soybean ERF gene *GmERF135* that was highly up-regulated by ethylene (ET), drought, salt, and low temperature treatments. Subcellular localization assay showed that the GmERF135 protein was targeted to the nucleus. Promoter *cis*-acting elements analysis suggested that numerous potential stress responsive *cis*-elements were distributed in the promoter region of *GmERF135*, including ABA-, light-, ET-, gibberellin (GA)-, and methyl jasmonate (MeJA)-responsive elements. Overexpression of *GmERF135* in *Arabidopsis* enhanced tolerance to drought and salt conditions. In addition, *GmERF135* promoted the growth of transgenic hairy roots under salt and exogenous ABA conditions. These results suggest that soybean GmERF135 may participate in both ABA and ET signaling pathways to regulate the responses to multiple stresses.

## Introduction

As a vital hormone in plants, abscisic acid (ABA) is essential to a myriad of aspects of plant growth and developmental processes, including plant embryo development, seed maturation, fruit maturity, and stomatal movement (Finkelstein et al., 2002). ABA signal transduction has been studied for many years (Pandey et al., 2006, 2009; Shen et al., 2006; Ma et al., 2009; Park et al., 2009; Yang and Guo, 2018), and the widely accepted molecular mechanism is that the pyrabactin resistant/PYR-like/regulatory component of ABA receptor (PYR/PYL/RCAR) acts as an ABA receptor which can bind to ABA, and then binds to and inhibits protein phosphatases type 2C (PP2Cs) (Santiago et al., 2009). In addition, the activity of SNF1-related protein kinases 2 (SnRK2s) is enhanced and can phosphorylate ABRE binding factors (AREB/ABFs) to induce physiological and biochemical changes in the process of ABA response (Hubbard et al., 2010; Yoshida et al., 2015). A recent study showed that ABFs can directly bind to the promoters of group A PP2C genes, and rapidly induce their expression on exogenous ABA treatments (Wang et al., 2018). However, the downstream molecular mechanism is yet not clearly understood.

Many transcription factors, such as MYC/MYB, bZIP/ABRE, AP2/ERF, and NAC, are regulated by ABA. The AP2/ERF family, the largest plant transcription factor family (Okamuro et al., 1997), can be divided into three subfamilies: the AP2, ERF, and RAV subfamilies. Among them, the ERF subfamily can specifically bind to GCC-box and/or the dehydration-responsive element/C-repeat (DRE/CRT) *cis*-acting elements (Allen et al., 1998).

The AP2/ERF family is involved in responses to various abiotic stresses and exogenous hormones (Xu et al., 2008, 2011; Klay et al., 2018). For example, overexpressing *TaERF1* enhances tolerance of physiological and environmental stresses, such as salt, drought, low temperature, exogenous ABA, ethylene (ET), salicylic acid (SA), and disease (Xu et al., 2007). Transgenic rice plants expressing *OsERF922* displayed higher susceptibility to *Magnaporthe oryzae* and NaCl compared to the wild type (WT), while the knockout mutant and RNAi lines enhanced resistance to these stresses (Liu et al., 2012). A recent study described how ABA modulates the expression level of ERF family, showing that the expression levels of ABA-responsive genes such as *RD22*, *LEA3*, and *PODs* were up-regulated after overexpressing *OsERF101* in rice, which enhanced its susceptibility to ABA (Jin et al., 2018).

Transcriptomic analysis of grapevine organs treated with or without ABA showed that ERF members were involved in differently expressed genes (DEGs), and the ERF subfamily had the most significant change compared to other transcription factors (Rattanakon et al., 2016). Although the ABA signal pathway has been extensively studied, it is yet unclear how regulation of the expression of downstream genes via ERF subfamily could enhance abiotic stress tolerances in soybean. To investigate whether the ERF subfamily is modulated by the ABA signal in soybean, we studied the response to exogenous ABA and identified the functions of *GmERF135* in transgenic *Arabidopsis* and soybean hairy roots.

## Materials and Methods

### Plant Materials and Treatment

Soybean cultivar “Tiefeng 8” was sown in pots containing vermiculite and grown at 25°C for 14 days and then treated with various abiotic stresses. For the various treatments, soybean plants were exposed in the air for rapid drought, dipped into 200 mM NaCl for salt stress, placed in 42°C/4°C chamber for high/low temperature stress, respectively. They were also placed in an airtight container filled with ET, or dipped in 100 μM ABA, 50 μM salicylic acid (SA), or 50 μM jasmonate (JA) for exogenous hormone stresses. Leaves of these plants were collected after 0, 0.5, 1, 2, 5, 12, and 24 h treatment and then immediately stored at −80°C for RNA extraction.

### Gene Structure and Protein Domain of ERFs in Soybean

The whole genome data of the candidate ERF genes was obtained from JGI Glyma1.0 annotation^1^ (Goodstein et al., 2012). Gene structure was analyzed by submitting the CDS of the candidate genes and whole genomic DNA sequences to the Gene Structure Display Server (GSDS) website^2^. Protein Fold Recognition Server (PHYRE2)^3^ was used for analysis of structural homology modeling of these genes, and DOG 2.0 was used to draw the protein domains.

### Quantitative Real-Time PCR (qRT-PCR)

Total RNA of the soybean plants was extracted using the TRIzol reagent (Invitrogen, Carlsbad, CA, United States). The specific primer pairs for the 16 genes were designed by Primer Premier 5.0 according to the cDNAs. qRT-PCR was conducted using the SYBR Premix Ex Taq^TM^ kit (TAKARA, Kusatsu, Japan) and the ABI Prism 7500 real-time PCR system (Thermo Fisher Scientific, Waltham, MA, United States). The 2^–ΔΔ^CT method was used to conduct qRT-PCR analysis (Le et al., 2011). Soybean Actin (U60506) was used as an internal control and sequence of specific primers were shown in Supplementary Table S1.

### Isolation and Promoter Analysis of *GmERF135*

The *GmERF135* gene was amplified by PCR and the primers for cloning the *GmERF135* were 5′-AATCATTATGTGTGGCGGTGCC-3′ and 5′-TATTCCTCGC TAATCGAAACTCCAGAG-3′. The PCR product was then cloned into the pEASY-T1 vector (TransGen, Beijing, China). 1,886 bp promotor region of *GmERF135* was cloned and submitted to PLACE^4^ and PlantCARE^5^ databases to analyze the putative *cis*-acting elements in the promoter region (Lescot et al., 2002).

### Subcellular Localization Analysis

The cDNA of *GmERF135* were augmented with PCR, connected to the N-terminus of *humanized green fluorescent protein* (*hGFP*) reporter gene under the control of the double Cauliflower Mosaic Virus (2 × CaMV) 35S promoter, and a recombinant plasmid was obtained (Scott et al., 1999). The recombinant plasmid was introduced into onion epidermal cells, while the onion epidermal cells with *hGFP* vector acted as the control. Fluorescence microscopy was used to identify *hGFP* expression (Xu et al., 2007; He et al., 2016).

### Generation of Transgenic *Arabidopsis* and Stress Treatments

AT5G47230 is the orthologue of *GmERF135* in *Arabidopsis*, which was named *ERF2* and used to investigate function in responses to various stresses. The seeds of two mutants, *erf2-1* and *erf2-2* (SALK_126889, SALK_076967) were mutated via T-DNA insertion.

The cDNA of *GmERF135* was obtained by using the specific primer pairs: 5′-TGATTACGCCAAGCTTATGTGTG GCGGTGCC-3′, 5′-CCGGGGATCCTCTAGAATCGAAACTC CAGAG-3′, and then were cloned into pBI121 under the control of the CaMV 35S promoter. The recombinant plasmid *35S::GmERF135* was sequenced and transformed into wild-type (WT) and the two mutants *Arabidopsis* lines using the vacuum infiltration method (Bechtold and Pelletier, 1998; Liu et al., 2013). T3 seeds of transgenic lines were selected for further analysis.

For root length assay, 30 seeds of each line were sown on 1/2-strength MS growth medium with or without 6% PEG, 75 mM NaCl, 1-aminocyclopropane-1-carboxylic acid (ACC), or in dark treatment for growth, respectively. At least 30 seedlings per line were randomly selected to measure root length. The cotyledon pieces of each line were recorded every 12 h. Each treatment contained three independent replicates.

### Soybean Hairy Root Induction and Stress Treatments

The *Superroot* of *Lotus corniculatus* and Cucumopine-type *Agrobacterium rhizogene* strain K599 with pGFPGUS*Plus* were provided by Professor Tian-Fu Han (CAAS, China). Seedling growth, rooting, hairy root induction, and hairy root transformation were performed as described by Chen et al. (2004, 2014)

GFP positive (GFP^+^) hairy roots were induced by K599 carrying the *p*GFPGUS*Plus*-*GmERF135* binary vector. These hairy roots were cultured on 1/2 MS medium supplemented with 0, 50, 85, 120, or 150 mM NaCl for salt treatment, and 50, 100, or 150 μM ABA for hormone treatment, respectively. They were then incubated at 24°C under a 16/8 h light/dark cycle condition for 2 weeks. After 24 h incubation at 105°C, the increase in dry weight (30 roots per unit) was measured and recorded. Each treatment contained three independent replicates.

## Results

### Molecular Characterization of Soybean Targeted ERF Genes

**FIGURE 1:**
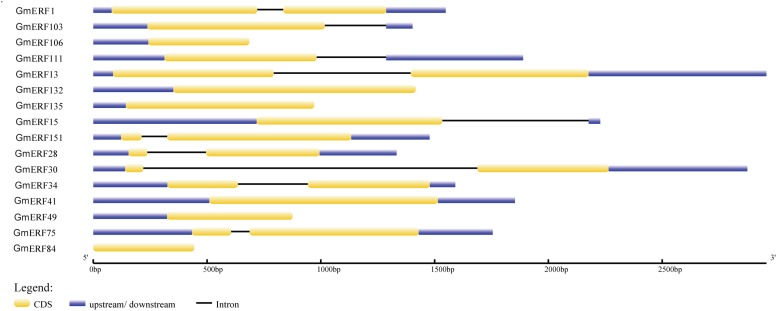
Intron-exon structure of candidate soybean ERF genes. Genome data for candidate ERF genes was obtained from JGI Glyma1.0 annotation. Intron-exon structure was produced using the GSDS online tool by submitting CDSs and genomic sequences of candidate genes. Yellow boxes represent exons, blue boxes represent introns, and black lines represent untranslated regions (UTRs).

**FIGURE 2:**
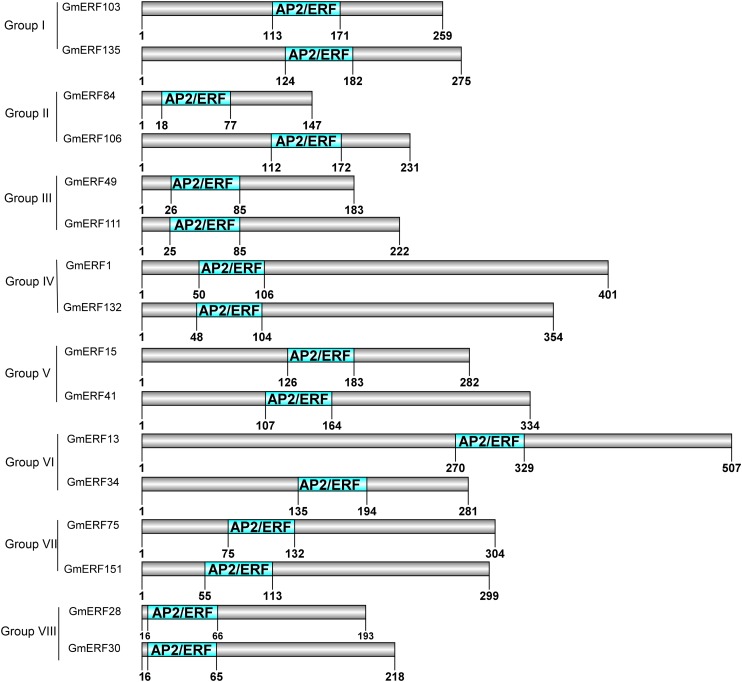
Protein domains of candidate soybean ERFs. Conserved motifs were analyzed using MEME, and DOG 2.0 was used to draw the domains. The conserved AP2/ERF domain is indicated by the green boxes. The number under green boxes shows the location of each domain.

In a previous study, 160 non-redundant soybean ERFs were identified using the Pfam database and SMART program, and these soybean ERFs were clustered into eight groups (Supplementary Table S2; Zhao et al., unpublished). To comprehensively understand the responses of soybean ERFs to ABA, 16 ERF genes were selected for further investigation according to the phylogenetic tree. Gene structure analysis showed that six genes had no introns, including *GmERF106*, *GmERF132*, *GmERF135*, *GmERF41*, *GmERF49*, and *GmERF84* (Figure 1). The remaining 10 ERF genes contained one intron, which was distributed in each cDNA region except for *GmERF103*, *GmERF111*, and *GmERF15*. The protein domains of each soybean ERF gene were drawn by DOG2.0 (Figure 2). All the proteins contained a conservative AP2/ERF domain, which was distributed in different positions of each protein sequence. The AP2/ERF domain of the proteins in each group displayed similar locations such as Group I, III, IV, V, VII, and VIII.

An expression pattern map of soybean ERFs was drawn based on the gene-chip data downloaded from the soybean genome database^6^. As shown in Supplementary Figure S1A, the soybean ERFs were expressed at different levels in various tissues and organs. Among them, *GmERF111* and *GmERF135* showed high expression levels in almost all tissues and organs. *GmERF75* showed high expression levels in root and nodule, and *GmERF49* was highly expressed in nodule and 10 DAF pod shell. *GmERF15* mainly expressed in young leaf. All the candidate genes had different expression patterns.

### Isolation and Subcellular Localization Characterization of *GmERF135*

**FIGURE 3:**
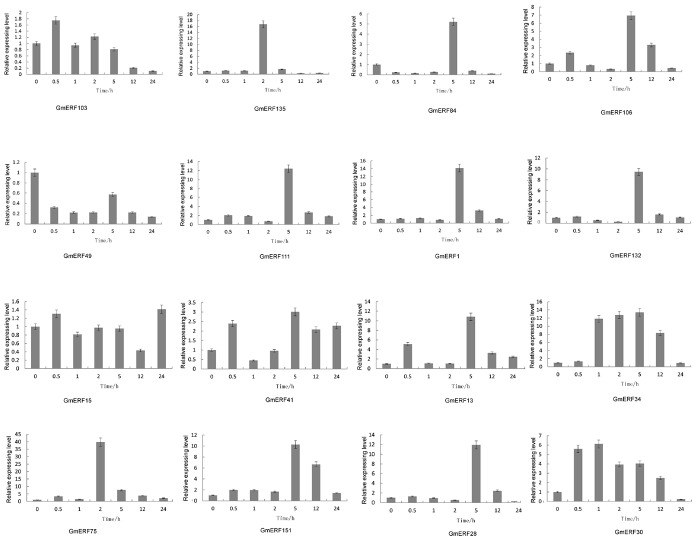
Relative expression levels of ERF genes after ABA treatment. Total RNA was extracted from soybean seedlings after ABA treatment for 0, 0.5, 1, 2, 5, 12, and 24 h and used for qRT-PCR. The data were shown as the means ± SD obtaining from three biological replicates.

Abscisic acid plays an important role in plant growth and development which is closely related to quality and yield in plants. To investigate how ABA affected the expression pattern of the 16 soybean ERF genes, qRT-PCR was conducted. Almost all the ERFs were up-regulated by ABA, except for *GmERF49* (Figure 3). The ERF gene *GmERF135* was the highest expressed gene after exogenous ABA treatment except for *GmERF75* which has been studied (Zhao et al., unpublished). The transcript level of *GmERF135* was activated and showed a 17-fold increase within 2 h after treatment and then declined to normal. Considering the high expression level and ABA-responsive increase in plants, *GmERF135* was selected for further study.

**FIGURE 4:**
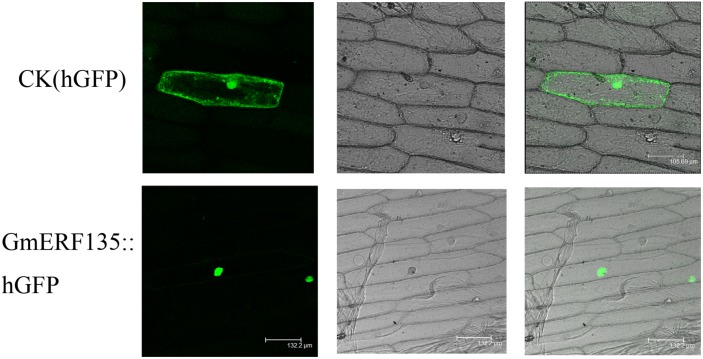
Subcellular localization of GmERF135. *GmERF135::hGFP* and control vector were introduced to onion epidermal cells and GmERF135::hGFP fusion protein and the control hGFP protein were expressed in the cells. Photographs were taken by confocal microscope (Leica) in the dark field for green fluorescence (A and D), in bright light for morphology of the cells (B and E) and in combination (C and F).

The subcellular localization assay was conducted to provide clues to understand intrinsic characteristics of cell activities. *hGFP* reporter gene fused to C-terminus of *GmERF135*, which could fluoresce when laser irradiated. The GmERF135::hGFP fused protein only fluoresced in nucleus, while the control fluoresced throughout the entire cell (Figure 4). This result showed that *GmERF135* functions in nucleus.

### *GmERF135* Promoter Region Comprises Diverse Stress-Responsive Elements

The promoter region is an important part of the gene which could regulate gene expression and control gene action. To investigate the potential regulation mechanism, the promoter region of *GmERF135*, which has 1886 bp length upstream of the start codon, was isolated. The PLACE and PlantCARE databases were used to analyze the putative regulatory elements in the promoter region. Several regulatory elements were identified to be involved in responses to abiotic stresses and plant hormones (Table 1).

**TABLE 1:** Analysis of putative *cis*-acting elements of *GmERF135* promoter.

***Cis*-acting element**	**Position**	**Strand**	**Matrix score**	**Sequences**	**Function**
ABRE	747	+	6	CACGTG	ABA responsive element
ABRE	1221	−	5	ACGTG	ABA responsive element
ABRE	748	+	5	ACGTG	ABA responsive element
Box 4	175	+	6	ATTAAT	Light responsive element
CGTCA-motif	1022	+	5	CGTCA	MeJA-responsive element
G-Box	747	+	6	CACGTG	Light responsive element
G-Box	1221	+	6	CACGTT	Light responsive element
MBS	1295	+	6	CAACTG	ABA and stress responsive element
TCT-motif	201	+	6	TCTTAC	light responsive element
TGACG-motif	1022	−	5	TGACG	MeJA responsive element
Core of MYBST1	57	−	5	GGATA	ABA and stress responsive element
Core of MYBST1	532	−	5	GGATA	ABA and stress responsive element
Core of MYBST1	1725	+	5	GGATA	ABA and stress responsive element
Core of MYBST1	1818	−	5	GGATA	ABA and stress responsive element
DPBF binding site	108	+	7	ACACNNG	ABA responsive element
DPBF binding site	134	+	7	ACACNNG	ABA responsive element
GARE	1,207	+	7	TAACAAR	Gibberellin responsive element
GARE	654	+	7	TAACAAR	Gibberellin responsive element
ERE	151	+	8	ATTTCAAA	Ethylene responsive element

The *GmERF135* promoter region contains many ABA and stress responsive elements, including ABA-responsive elements (ABREs, 3 hits), MYBST1 core sequences (4 hits), MYB binding sites (MBS, 1 hit), and DPBF binding sites (2 hits) (Table 1). Except for ABREs, three hormone-responsive elements were predicted, including an ethylene- responsive element (ERE, 1 hit), MeJA-responsive element TGACG-motif (1 hit) and CGTCA-motif (1 hit), and GA-responsive element (GARE, 2 hits). In addition, TCT-motif (1 hit), G-box (2 hits), and Box 4 (1 hit) were found, which could act as light-responsive elements in the promoter region of *GmERF135*. These elements suggested that *GmERF135* may be involved in responses to multiple abiotic stresses and exogenous hormones.

### The Roles of *GmERF135* in Responding to Multiple Stimuli

**FIGURE 5:**
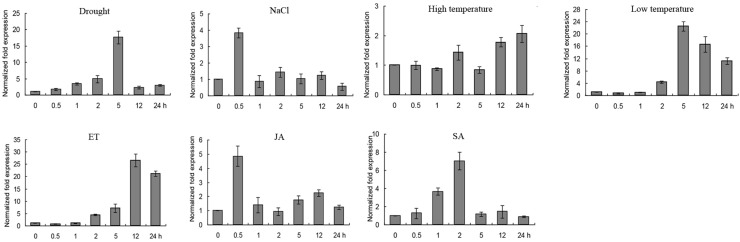
Expression patterns of *GmERF135* under multiple stimuli. Two-week soybean seedlings were given various abiotic stresses and exogenous hormones for 0, 0.5, 1, 2, 5, 12, and 24 h, which were used to extract RNA and obtained cDNA. The transcript levels of *GmERF135* after multiple stresses were quantified by qRT-PCR, including drought, NaCl, high/low temperature, ET (ethylene), JA (jasmonate), and SA (salicylic acid). Data were shown referring to three biological replicates.

Quantitative Real-Time PCR was conducted to investigate the expression level of *GmERF135* under abiotic stresses, such as drought, salt, high/low temperature, and exogenous hormones including ET, SA, and JA. *GmERF135* was induced by almost all the abiotic stresses and exogenous hormones. Remarkably, the expression level of the gene was extremely up-regulated by drought, low temperature, and ET treatment. The transcription of *GmERF135* peaked at 5 h under drought or low-temperature treatment which had 18-fold / 22-fold increases, respectively (Figure 5). The peak of *GmERF135* transcription appeared at 12 h after treatment with ET, which showed a 28-fold increase. In contrast, *GmERF135* rapidly reached the maximum transcript level within 0.5 h under the NaCl and JA treatments, and then declined to baseline after 1 h. Under the SA treatment, *GmERF135* was induced and reached the peak within 2 h (about 7-fold) and then declined to normal level in 24 h. These results showed that *GmERF135* may be involved in responses to multiple stimuli.

### *GmERF135* Rescued Two *erf2* Mutants and Affected Growth of the Root and Cotyledon

Total RNA was extracted for semi-quantitative PCR from hypocotyls, root, stem, and leaf tissues of normal growth soybean seedlings. Actin primers were used as a parallel reaction to normalize the added template amounts. *GmERF135* was predominantly expressed in the leaf, less in the hypocotyl and stem, and very little in the root (Supplementary Figure S1B).

**FIGURE 6:**
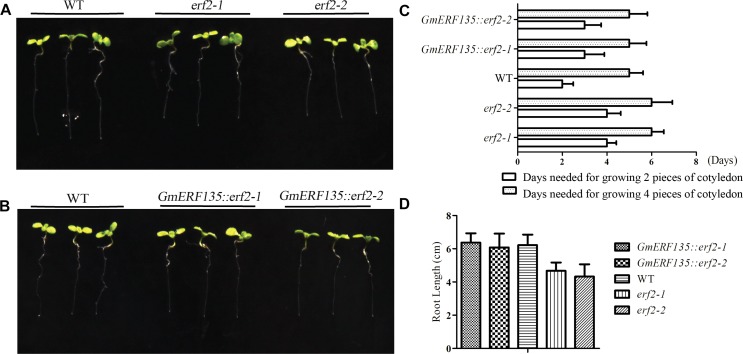
*GmERF135* rescued the speed of cotyledon growth in the two mutants. **(A)** Phenotype of two *erf2* mutants and WT *Arabidopsis* grown on the MS solid medium. **(B)** Phenotype of two *GmERF135::erf2* lines and WT *Arabidopsis* grown on 1/2 MS solid medium. **(C)** Days needed for growing the cotyledon. Statistic data were recorded every 12 h. Three independent experiments (20 plants each) were performed. Error bars indicate SD of three biological replicates (*n* = 3). **(D)** Average root lengths of WT, *erf2*, and *GmERF135::erf2* after grown on 1/2 MS medium for 6 days. Data were shown as the means ± SD of three biological replicates (at least 30 individuals per line).

To assess whether *GmERF135* could rescue the two mutants, the seeds of transgenic *GmERF135::erf2* lines, WT, and two *erf2* mutants were sown on the 1/2 MS medium for growth and the plant growth rate was recorded each 12 h. Six day later, the phenotypes were imaged (Figures 6A,B).

Growth rate assay showed *GmERF135::erf2* lines displayed faster growth rate compared to the mutants (Figure 6C). WT plants required 2 d of growth to sprout the two pieces of the cotyledon while the two mutants required 4 days. After introduction of *GmERF135* in *erf2* lines, 3 days was required for the same process. The two mutants needed 6 days to reach 4-leaf stage, while the transgenic *GmERF135::erf2* lines had similar growth rate with WT, which only needed 5 d. Root length assay showed there is an approximately 25% decrease in *erf2* mutants compared to WT lines (Figure 6D). After overexpressing *GmERF135* in the *erf2* mutants, the lines had similar root lengths to WT and the phenotype of which was rescued. These results showed that overexpression of *GmERF135* in the two *erf2* mutants partly rescued two *erf2* mutants and affected growth of root and cotyledon in *Arabidopsis*.

### *GmERF135* Promotes Plant Growth Under Drought and Salt Stresses in *Arabidopsis*

**FIGURE 7:**
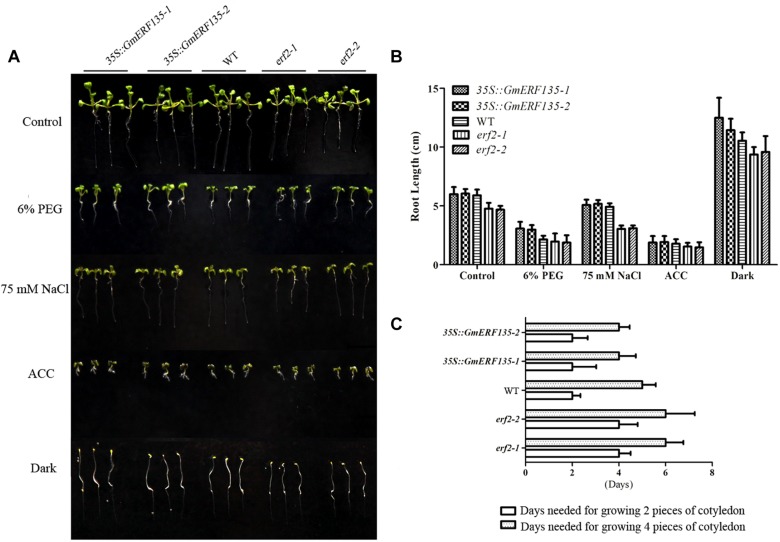
*GmERF135* transgenic *Arabidopsis* lines improved cotyledon growth under the NaCl and PEG conditions. **(A)** Phenotype of *GmERF135* transgenic *Arabidopsis* lines under various stresses. Three-day-old *GmERF135* transgenic *Arabidopsis* lines were transferred to 1/2 MS medium with 6% PEG, 75 mM NaCl, ACC, or grown in darkness. **(B)** Average root lengths of WT, *erf2*, and *35S::GmERF135* lines. Three-day-old seedlings were treated with various stresses for 3 days and the data were shown as the means ± SD of three biological replicates (at least 30 individuals per line). **(C)** Days needed for growing cotyledon. Statistic data were recorded every 12 h. Three biological replicates were performed.

To determine whether *GmERF135* confers abiotic stress tolerance to *Arabidopsis* plants, 3-day-old WT, *erf2* mutants, and *GmERF135* overexpression seedlings were transferred to 1/2 MS medium with or without 6% PEG, 75 mM NaCl, ACC, and dark environment grown for 3 days. The growth rate and the root length of 30 seedlings in each line were recorded (Figures 7B,C). Statistics showed that the average root length of *erf2* was shorter than the WT and transgenic *Arabidopsis* plants under all conditions except for ACC treatment (Figures 7A,B). The transgenic plants displayed larger cotyledon than the WT and mutants under NaCl and PEG treatments, which suggested that *GmERF135* enhanced the growth rate of *Arabidopsis* plants. Overexpression of *GmERF135* in *Arabidopsis* resulted in a longer root compared to the WT and two *erf2* mutants under 6% PEG, 75 mM NaCl, and dark conditions. No detectable difference was observed between the transgenic lines and WT under ACC conditions.

To further understood the response to various stresses in *Arabidopsis*, some marker genes, such as *NCED3* (Ruggiero et al., 2004), *RD29A* (Yamaguchi-Shinozaki and Shinozaki, 1994), *COR15A* (Xu et al., 2011), *DREB2A* (Liu et al., 1998), *ABA1*, *ABA2* (He et al., 2016), *ACO4*, and *ACS2* (Kim et al., 2013) were selected for qRT-PCR (Supplementary Figure S2).

### *GmERF135* Improves Tolerance of Salt and ABA in Soybean

**FIGURE 8:**
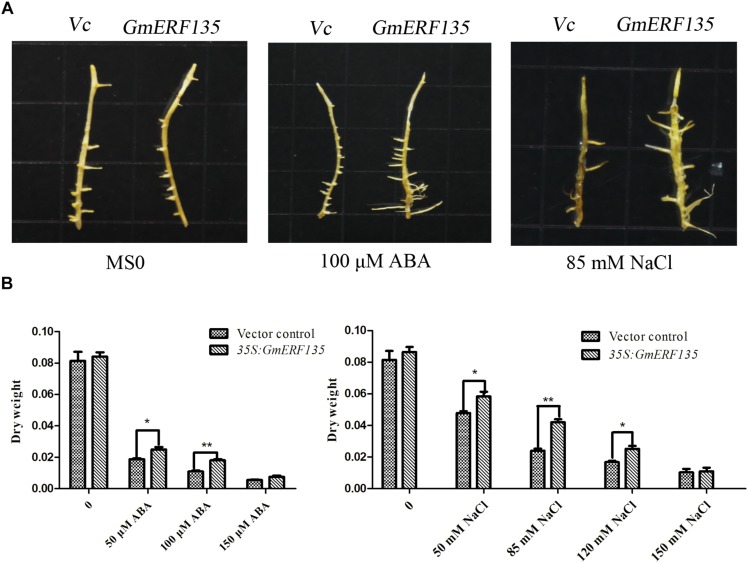
*GmERF135* transgenic soybean hairy roots enhanced tolerance to ABA and salt. **(A)** Phenotypes of WT and *GmERF135* transgenic soybean hairy roots grown on MS medium with different concentrations of ABA and NaCl. Vc for Vector control. **(B)** Dry weight of WT and transgenic soybean hairy roots after 2 weeks of treatment. Data are shown as means ± SD of three biological experiments. Student’s *t* test was used for statistical analysis and asterisks indicate significant differences from the vector control at ^*^P < 0.05 and ^∗∗^*P* < 0.01.

To investigate the roles of *GmERF135* in soybean, the pGUS-*GmERF135* expression vector was constructed and transformed into soybean hairy roots, which were studied on MS basal medium supplemented with different concentrations of ABA and NaCl. *GmERF135* transgenic soybean hairy roots experienced greater growth under the treatments compared to the vector control, especially under the 100 μM ABA and 85 mM NaCl conditions (Figure 8A).

Dry weight measurement results showed that *GmERF135* hairy roots increased by about 10% compared to the vector under the MS0 condition, while transgenic hairy roots displayed a 6.24–75% improvement compared to WT under NaCl and exogenous ABA at the different concentrations (Figure 8B). For NaCl treatment, there has a 6.24–75.03% increase in the average dry weight of hairy roots overexpressing *GmERF135*. For exogenous ABA, the transgenic hairy roots displayed a 36–65% improvement compared to vector control under exogenous ABA treatment at different concentrations (Figure 8B). It is noteworthy that a total of 64.46% improvement in *GmERF135* hairy roots was observed under the 100 μM ABA condition, and an improvement of 75.03% with 85 mM NaCl (Figure 8B). Results of dry weight measurements confirmed the conclusion that *GmERF135* could promote plant growth under ABA and NaCl conditions.

## Discussion

Abscisic acid is well-known to be involved in responses to multiple stresses, such as drought, salt, and cold, and to induce expression of stress-related genes in plants (Leung et al., 1997; Rohde et al., 2000; Rook et al., 2001; Xu et al., 2007). The interaction of the ABA and ET signal pathway is extremely complex and intricate. AREB/ABFs, members of the bZIP family of transcription factors, participate in the ABA signal pathway which can specifically bind to ABRE *cis*-element to modulate the expression of downstream target genes (Bonetta and McCourt, 1998; Leung and Giraudat, 1998; Li et al., 2012). Fujita et al. (2005) observed that *AREB1* can act as a transcription activator in ABRE-dependent ABA signaling, which enhances drought tolerance in *Arabidopsis*. Overexpression of the *AREB1* gene in *Arachis hypogaea* could enhance drought tolerance by modulating ROS scavenging and maintaining content (Li et al., 2013). Phosphorylated AREB/ABFs may bind to the ABRE *cis*-element of *GmERF135* promotor region to activate its transcription level (Fujita et al., 2013), which could activate or repress the transcription of targeted genes. In our study, analysis of the *GmERF135* promoter region showed that three ABREs were located at the promoter region, which may be binding sites of AREB/ABFs to affect the transcript of *GmERF135*. GmERF135 has a conversed AP2/ERF domain, which could specifically bind to GCC-box and/or dehydration-responsive element/C-repeat (DRE/CRT) *cis*-acting elements to modulate the expression of pathogenesis- and abiotic stresses-related genes (Hao et al., 1998; Chakravarthy et al., 2003; Zarei et al., 2011).

Previous studies showed there is an antagonistic interaction between ABA and JA-ET signaling pathways ABA for disease response (Beaudoin et al., 2000; Anderson et al., 2004; Martin-Rodriguez et al., 2011). A recent study showed that *SlAREB1* could enhance the expression of ethylene biosynthetic genes *ACS* and *ACO* in tomato fruits, which are the two key genes of ET synthesis (Mou et al., 2018). In our study, qRT-PCR analysis showed that the transcription of both *AtACO4* and *AtACS2* increased (Supplementary Figure S2) in transgenic *Arabidopsis* lines, which suggested acceleration of ET production. ET acceleration could trigger a series of reactions of ET and activate the transcript of ERF responsive genes (Guo and Ecker, 2004). At the same time, the transcript levels of *ABA1* and *ABA2*, key factors of ABA synthesis, were also upregulated after overexpressing *GmERF135* in *Arabidopsis* lines. These results suggested that GmERF135 may participate in both ET and ABA signaling pathways, and the regulation between the two signaling pathways needs further research.

Except for ABREs, other stress-related elements were also distributed in the promotor region of *GmERF135*, such as the MYBST1 core sequence, MBS, and ERE (Table 1). The MYBST1 core sequence and MBS elements have been demonstrated to be involved in drought, low temperature, salt, and ABA stress responses (Abe et al., 2003). qRT-PCR showed that *GmERF135* is induced by multiple stresses, including drought, salt, low temperature, exogenous ET, SA, and JA. These changes may be caused by some corresponding *cis*-elements in promoter regions which can be bound by specific transcription factors (Huang et al., 2012; Buscaill and Rivas, 2014). These results suggest that soybean ERF gene *GmERF135* is a key factor which participates in multiple signaling pathways to regulate expression levels of stress-related genes.

## Author Contributions

Z-SX and Y-ZM coordinated the project, conceived and designed experiments, and edited the manuscript. M-JZ and L-JY conducted the bioinformatic work, performed experiments and wrote the first draft. JM revised the manuscript and figures. J-CZ conducted the bioinformatic work. Y-XW, J-HL, and J-DF contributed with valuable discussions. MC provided analytical tools and managed reagents. All authors have read and approved the final manuscript.

## Conflict of Interest Statement

The authors declare that the research was conducted in the absence of any commercial or financial relationships that could be construed as a potential conflict of interest.

## Footnotes

1 https://phytozome.jgi.doe.gov/pz/portal.html

2 http://gsds.cbi.pku.edu.cn/

3 http://www.sbg.bio.ic.ac.uk/phyre2/html/page.cgi?id=index

4 http://www.dna.affrc.go.jp/PLACE/

5 http://bioinformatics.psb.ugent.be/webtools/plantcare/html/

6 https://soybase.org/soyseq

## Supplementary Material

FIGURE S1 Expression patterns of candidate soybean ERFs in different organs. **(A)** Expression patterns of candidate soybean ERFs in different organs. Normalized expression data for the soybean ERF genes were collected from the SoyBase (http://www.soybase.org/). Expression (vertical coordinates) is in transcripts per million (TPM). **(B)** Semi-quantitative PCR of *GmERF135* in different organs.

FIGURE S2 Express levels of various stresses-related genes after treatment. Two-week-old WT and transgenic *Arabidopsis* lines were used to extract total RNA. GmActin/AtActin was used for normalization. Data were shown as the means ± SDs of three experiments.

TABLE S1 Specific primers of each gene for qRT-PCR. All the primers were designed via Primer Primer 5.0.

TABLE S2 Classification of 160 soybean ERF genes.

## References

[B1] Abe H. Urao T. Ito T. Seki M. Shinozaki K. Yamaguchi-Shinozaki K. (2003). *Arabidopsis* AtMYC2 (bHLH) and AtMYB2 (MYB) function as transcriptional activators in abscisic acid signaling. *Plant Cell* 15 63–78. 10.1105/tpc.006130 12509522 PMC143451

[B2] Allen M. D. Yamasaki K. Ohme-Takagi M. Tateno M. Suzuki M. (1998). A novel mode of DNA recognition by a beta-sheet revealed by the solution structure of the GCC-box binding domain in complex with DNA. *EMBO J.* 17 5484–5496. 10.1093/emboj/17.18.5484 9736626 PMC1170874

[B3] Anderson J. P. Badruzsaufari E. Schenk P. M. Manners J. M. Desmond O. J. Ehlert C. (2004). Antagonistic interaction between abscisic acid and jasmonate-ethylene signaling pathways modulates defense gene expression and disease resistance in *Arabidopsis*. *Plant Cell* 16 3460–3479. 10.1105/tpc.104.025833 15548743 PMC535886

[B4] Beaudoin N. Serizet C. Gosti F. Giraudat J. (2000). Interactions between abscisic acid and ethylene signaling cascades. *Plant Cell* 12 1103–1115. 10.1105/tpc.12.7.1103 10899977 PMC149052

[B5] Bechtold N. Pelletier G. (1998). In planta agrobacterium-mediated transformation of adult Arabidopsis thaliana plants by vacuum infiltration. *Methods Mol. Biol.* 82 259–266. 10.1385/0-89603-391-0:259 9664431

[B6] Bonetta D. McCourt P. (1998). Genetic analysis of ABA signal transduction pathways. *Trends Plant Sci.* 3 231–235. 10.1016/s1360-1385(98)01241-2

[B7] Buscaill P. Rivas S. (2014). Transcriptional control of plant defence responses. *Curr. Opin. Plant Biol.* 20 35–46. 10.1016/j.pbi.2014.04.004 24840291

[B8] Chakravarthy S. Tuori R. P. D’Ascenzo M. D. Fobert P. R. Despres C. Martin G. B. (2003). The tomato transcription factor Pti4 regulates defense-related gene expression via GCC box and non-GCC box cis elements. *Plant Cell* 15 3033–3050. 10.1105/tpc.017574 14630974 PMC282854

[B9] Chen L. Jiang B. Wu C. Sun S. Hou W. Han T. (2014). *GmPRP2* promoter drives root-preferential expression in transgenic *Arabidopsis* and soybean hairy roots. *BMC Plant Biol.* 14:245. 10.1186/s12870-014-0245-z 25224536 PMC4172956

[B10] Chen M. Chory J. Fankhauser C. (2004). Light signal transduction in higher plants. *Annu. Rev. Genet.* 38 87–117. 10.1146/annurev.genet.38.072902.092259 15568973

[B11] Finkelstein R. R. Gampala S. S. L. Rock C. D. (2002). Abscisic acid signaling in seeds and seedlings. *Plant Cell* 14 S15–S45. 12045268 10.1105/tpc.010441 PMC151246

[B12] Fujita Y. Fujita M. Satoh R. Maruyama K. Parvez M. M. Seki M. (2005). AREB1 is a transcription activator of novel ABRE-dependent ABA signaling that enhances drought stress tolerance in *Arabidopsis*. *Plant Cell* 17 3470–3488. 10.1105/tpc.105.035659 16284313 PMC1315382

[B13] Fujita Y. Yoshida T. Yamaguchi-Shinozaki K. (2013). Pivotal role of the AREB/ABF-SnRK2 pathway in ABRE-mediated transcription in response to osmotic stress in plants. *Physiol. Plant.* 147 15–27. 10.1111/j.1399-3054.2012.01635.x 22519646

[B14] Goodstein D. M. Shu S. Howson R. Neupane R. Hayes R. D. Fazo J. (2012). Phytozome: a comparative platform for green plant genomics. *Nucleic Acids Res.* 40 D1178–D1186. 10.1093/nar/gkr944 22110026 PMC3245001

[B15] Guo H. Ecker J. R. (2004). The ethylene signaling pathway: new insights. *Curr. Opin. Plant Biol.* 7 40–49. 10.1016/j.pbi.2003.11.011 14732440

[B16] Hao D. Ohme-Takagi M. Sarai A. (1998). Unique mode of GCC box recognition by the DNA-binding domain of ethylene-responsive element-binding factor (ERF domain) in plant. *J. Biol. Chem.* 273 26857–26861. 10.1074/jbc.273.41.26857 9756931

[B17] He G. H. Xu J. Y. Wang Y. X. Liu J. M. Li P. S. Chen M. (2016). Drought-responsive WRKY transcription factor genes TaWRKY1 and TaWRKY33 from wheat confer drought and/or heat resistance in *Arabidopsis*. *BMC Plant Biol.* 16:116. 10.1186/s12870-016-0806-4 27215938 PMC4877946

[B18] Huang G. T. Ma S. L. Bai L. P. Zhang L. Ma H. Jia P. (2012). Signal transduction during cold, salt, and drought stresses in plants. *Mol. Biol. Rep.* 39 969–987. 10.1007/s11033-011-0823-1 21573796

[B19] Hubbard K. E. Nishimura N. Hitomi K. Getzoff E. D. Schroeder J. I. (2010). Early abscisic acid signal transduction mechanisms: newly discovered components and newly emerging questions. *Genes Dev.* 24 1695–1708. 10.1101/gad.1953910 20713515 PMC2922499

[B20] Jin Y. Pan W. Y. Zheng X. F. Cheng X. Liu M. M. Ma H. (2018). OsERF101, an ERF family transcription factor, regulates drought stress response in reproductive tissues. *Plant Mol. Biol.* 98 51–65. 10.1007/s11103-018-0762-5 30143992

[B21] Kim J.-G. Stork W. Mudgett M. B. (2013). *Xanthomonas* type III effector XopD desumoylates tomato transcription factor SlERF4 to suppress ethylene responses and promote pathogen growth. *Cell Host Microbe* 13 143–154. 10.1016/j.chom.2013.01.006 23414755 PMC3622456

[B22] Klay I. Gouia S. Lu M. Mila I. Khoudi H. Bernadac A. (2018). Ethylene response factors (ERF) are differentially regulated by different abiotic stress types in tomato plants. *Plant Sci.* 274 137–145. 10.1016/j.plantsci.2018.05.023 30080597

[B23] Le D. T. Nishiyama R. Watanabe Y. Mochida K. Yamaguchi-Shinozaki K. Shinozaki K. (2011). Genome-wide expression profiling of soybean two-component system genes in soybean root and shoot tissues under dehydration stress. *DNA Res.* 18 17–29. 10.1093/dnares/dsq032 21208938 PMC3041507

[B24] Lescot M. Dehais P. Thijs G. Marchal K. Moreau Y. Van de Peer Y. (2002). PlantCARE, a database of plant cis-acting regulatory elements and a portal to tools for in silico analysis of promoter sequences. *Nucleic Acids Res.* 30 325–327. 10.1093/nar/30.1.325 11752327 PMC99092

[B25] Leung J. Giraudat J. (1998). Cloning genes of *Arabidopsis thaliana* by chromosome walking. *Methods Mol. Biol.* 82 277–303. 10.1385/0-89603-391-0:277 9664433

[B26] Leung J. Merlot S. Giraudat J. (1997). The *Arabidopsis* ABSCISIC ACID-INSENSITIVE2 (ABI2) and ABI1 genes encode homologous protein phosphatases 2C involved in abscisic acid signal transduction. *Plant Cell* 9 759–771. 10.1105/tpc.9.5.759 9165752 PMC156954

[B27] Li W. Cui X. Meng Z. L. Huang X. H. Xie Q. Wu H. (2012). Transcriptional regulation of Arabidopsis MIR168a and argonaute1 homeostasis in abscisic acid and abiotic stress responses. *Plant Physiol.* 158 1279–1292. 10.1104/pp.111.188789 22247272 PMC3291255

[B28] Li X. Y. Liu X. Yao Y. Li Y. H. Liu S. He C. Y. (2013). Overexpression of *Arachis hypogaea* AREB1 gene enhances drought tolerance by modulating ROS scavenging and maintaining endogenous ABA content. *Int. J. Mol. Sci.* 14 12827–12842. 10.3390/ijms140612827 23783278 PMC3709814

[B29] Liu D. F. Chen X. J. Liu J. Q. Ye J. C. Guo Z. J. (2012). The rice ERF transcription factor OsERF922 negatively regulates resistance to *Magnaporthe oryzae* and salt tolerance. *J. Exp. Bot.* 63 3899–3911. 10.1093/jxb/ers079 22442415 PMC3388842

[B30] Liu P. Xu Z. S. Pan-Pan L. Hu D. Chen M. Li L. C. (2013). A wheat PI4K gene whose product possesses threonine autophophorylation activity confers tolerance to drought and salt in *Arabidopsis*. *J. Exp. Bot.* 64 2915–2927. 10.1093/jxb/ert133 23682116 PMC3741686

[B31] Liu Q. Kasuga M. Sakuma Y. Abe H. Miura S. Yamaguchi-Shinozaki K. (1998). Two transcription factors, DREB1 and DREB2, with an EREBP/AP2 DNA binding domain separate two cellular signal transduction pathways in drought- and low-temperature-responsive gene expression, respectively, in *Arabidopsis*. *Plant Cell* 10 1391–1406. 10.1105/tpc.10.8.1391 9707537 PMC144379

[B32] Ma Y. Szostkiewicz I. Korte A. Moes D. Yang Y. Christmann A. (2009). Regulators of PP2C phosphatase activity function as abscisic acid sensors. *Science* 324 1064–1068. 10.1126/science.1172408 19407143

[B33] Martin-Rodriguez J. A. Leon-Morcillo R. Vierheilig H. Ocampo J. A. Ludwig-Muller J. Garcia-Garrido J. M. (2011). Ethylene-dependent/ethylene-independent ABA regulation of tomato plants colonized by arbuscular mycorrhiza fungi. *New Phytol.* 190 193–205. 10.1111/j.1469-8137.2010.03610.x 21232061

[B34] Mou W. Li D. Luo Z. Li L. Mao L. Ying T. (2018). SlAREB1 transcriptional activation of NOR is involved in abscisic acid-modulated ethylene biosynthesis during tomato fruit ripening. *Plant Sci.* 276 239–249. 10.1016/j.plantsci.2018.07.015 30348324

[B35] Okamuro J. K. Caster B. Villarroel R. Van Montagu M. Jofuku K. D. (1997). The AP2 domain of APETALA2 defines a large new family of DNA binding proteins in *Arabidopsis*. *Proc. Natl. Acad. Sci. U.S.A.* 94 7076–7081. 10.1073/pnas.94.13.7076 9192694 PMC21287

[B36] Pandey S. Chen J. G. Jones A. M. Assmann S. M. (2006). G-protein complex mutants are hypersensitive to abscisic acid regulation of germination and postgermination development. *Plant Physiol.* 141 243–256. 10.1104/pp.106.079038 16581874 PMC1459317

[B37] Pandey S. Nelson D. C. Assmann S. M. (2009). Two novel GPCR-type G proteins are abscisic acid receptors in *Arabidopsis*. *Cell* 136 136–148. 10.1016/j.cell.2008.12.026 19135895

[B38] Park S. Y. Fung P. Nishimura N. Jensen D. R. Fujii H. Zhao Y. (2009). Abscisic acid inhibits type 2C protein phosphatases via the PYR/PYL family of START proteins. *Science* 324 1068–1071. 10.1126/science.1173041 19407142 PMC2827199

[B39] Rattanakon S. Ghan R. Gambetta G. A. Deluc L. G. Schlauch K. A. Cramer G. R. (2016). Abscisic acid transcriptomic signaling varies with grapevine organ. *BMC Plant Biol.* 16:72. 10.1186/s12870-016-0763-y 27001301 PMC4802729

[B40] Rohde A. Kurup S. Holdsworth M. (2000). ABI3 emerges from the seed. *Trends Plant Sci.* 5 418–419. 10.1016/s1360-1385(00)01736-2 11203275

[B41] Rook F. Corke F. Card R. Munz G. Smith C. Bevan M. W. (2001). Impaired sucrose-induction mutants reveal the modulation of sugar-induced starch biosynthetic gene expression by abscisic acid signalling. *Plant J.* 26 421–433. 10.1046/j.1365-313x.2001.2641043.x 11439129

[B42] Ruggiero B. Koiwa H. Manabe Y. Quist T. M. Inan G. Saccardo F. (2004). Uncoupling the effects of abscisic acid on plant growth and water relations. Analysis of sto1/nced3, an abscisic acid-deficient but salt stress-tolerant mutant in *Arabidopsis*. *Plant Physiol.* 136 3134–3147. 10.1104/pp.104.046169 15466233 PMC523374

[B43] Santiago J. Rodrigues A. Saez A. Rubio S. Antoni R. Dupeux F. (2009). Modulation of drought resistance by the abscisic acid receptor PYL5 through inhibition of clade A PP2Cs. *Plant J.* 60 575–588. 10.1111/j.1365-313X.2009.03981.x 19624469

[B44] Scott A. Wyatt S. Tsou P. L. Robertson D. Allen N. S. (1999). Model system for plant cell biology: GFP imaging in living onion epidermal cells. *Biotechniques* 26 1125, 1128–1132. 10.2144/99266st04 10376152

[B45] Shen Y. Y. Wang X. F. Wu F. Q. Du S. Y. Cao Z. Shang Y. (2006). The Mg-chelatase H subunit is an abscisic acid receptor. *Nature* 443 823–826. 10.1038/nature05176 17051210

[B46] Wang X. Guo C. Peng J. Li C. Wan F. Zhang S. (2018). ABRE-BINDING FACTORS play a role in the feedback regulation of ABA signaling by mediating rapid ABA induction of ABA co-receptor genes. *New Phytol.* 221 341–355. 10.1111/nph.15345 30019753

[B47] Xu Z. S. Chen M. Li L. C. Ma Y. Z. (2008). Functions of the ERF transcription factor family in plants. *Bot. Botanique* 86 969–977. 10.1139/b08-041

[B48] Xu Z. S. Chen M. Li L. C. Ma Y. Z. (2011). Functions and application of the AP2/ERF transcription factor family in crop improvement. *J. Integr. Plant Biol.* 53 570–585. 10.1111/j.1744-7909.2011.01062.x 21676172

[B49] Xu Z. S. Xia L. Q. Chen M. Cheng X. G. Zhang R. Y. Li L. C. (2007). Isolation and molecular characterization of the *Triticum aestivum* L. ethylene-responsive factor 1 (TaERF1) that increases multiple stress tolerance. *Plant Mol. Biol.* 65 719–732. 10.1007/s11103-007-9237-9 17874224

[B50] Yamaguchi-Shinozaki K. Shinozaki K. (1994). A novel cis-acting element in an *Arabidopsis* gene is involved in responsiveness to drought, low-temperature, or high-salt stress. *Plant Cell* 6 251–264. 10.1105/tpc.6.2.251 8148648 PMC160431

[B51] Yang Y. Guo Y. (2018). Unraveling salt stress signaling in plants. *J. Integr. Plant Biol.* 60 796–804. 10.1111/jipb.12689 29905393

[B52] Yoshida T. Fujita Y. Maruyama K. Mogami J. Todaka D. Shinozaki K. (2015). Four *Arabidopsis* AREB/ABF transcription factors function predominantly in gene expression downstream of SnRK2 kinases in abscisic acid signalling in response to osmotic stress. *Plant Cell Environ.* 38 35–49. 10.1111/pce.12351 24738645 PMC4302978

[B53] Zarei A. Korbes A. P. Younessi P. Montiel G. Champion A. Memelink J. (2011). Two GCC boxes and AP2/ERF-domain transcription factor ORA59 in jasmonate/ethylene-mediated activation of the PDF1.2 promoter in *Arabidopsis*. *Plant Mol. Biol.* 75 321–331. 10.1007/s11103-010-9728-y 21246258 PMC3044237

