# Supplementary figures and images for: The Roles of GmERF135 in Improving Salt Tolerance and Decreasing ABA Sensitivity in Soybean
Source: Front Plant Sci. 2019 Jul 23;10:940. doi: 10.3389/fpls.2019.00940 (PMC6664033; doi:10.3389/fpls.2019.00940)

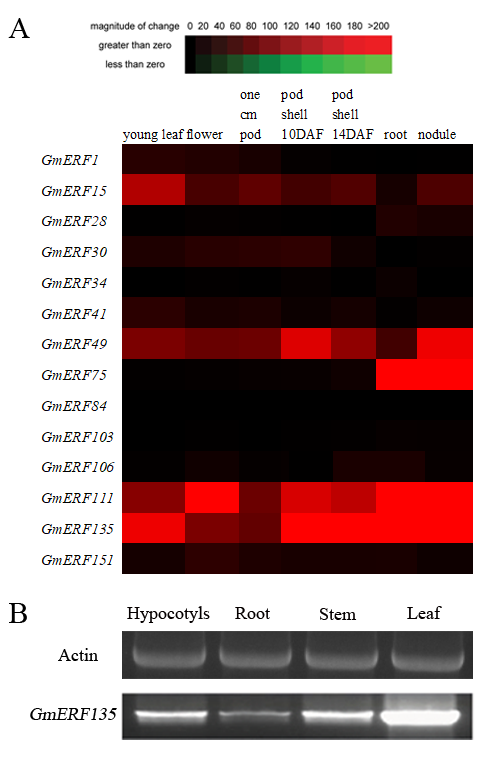

Supplement: FIGURE S1 — Expression patterns of candidate soybean ERFs in different organs. (A) Expression patterns of candidate soybean ERFs in different organs. Normalized expression data for the soybean ERF genes were collected from the SoyBase (http://www.soybase.org/). Expression (vertical coordinates) is in transcripts per million (TPM). (B) Semi-quantitative PCR of GmERF135 in different organs. [file Image_1.TIF]

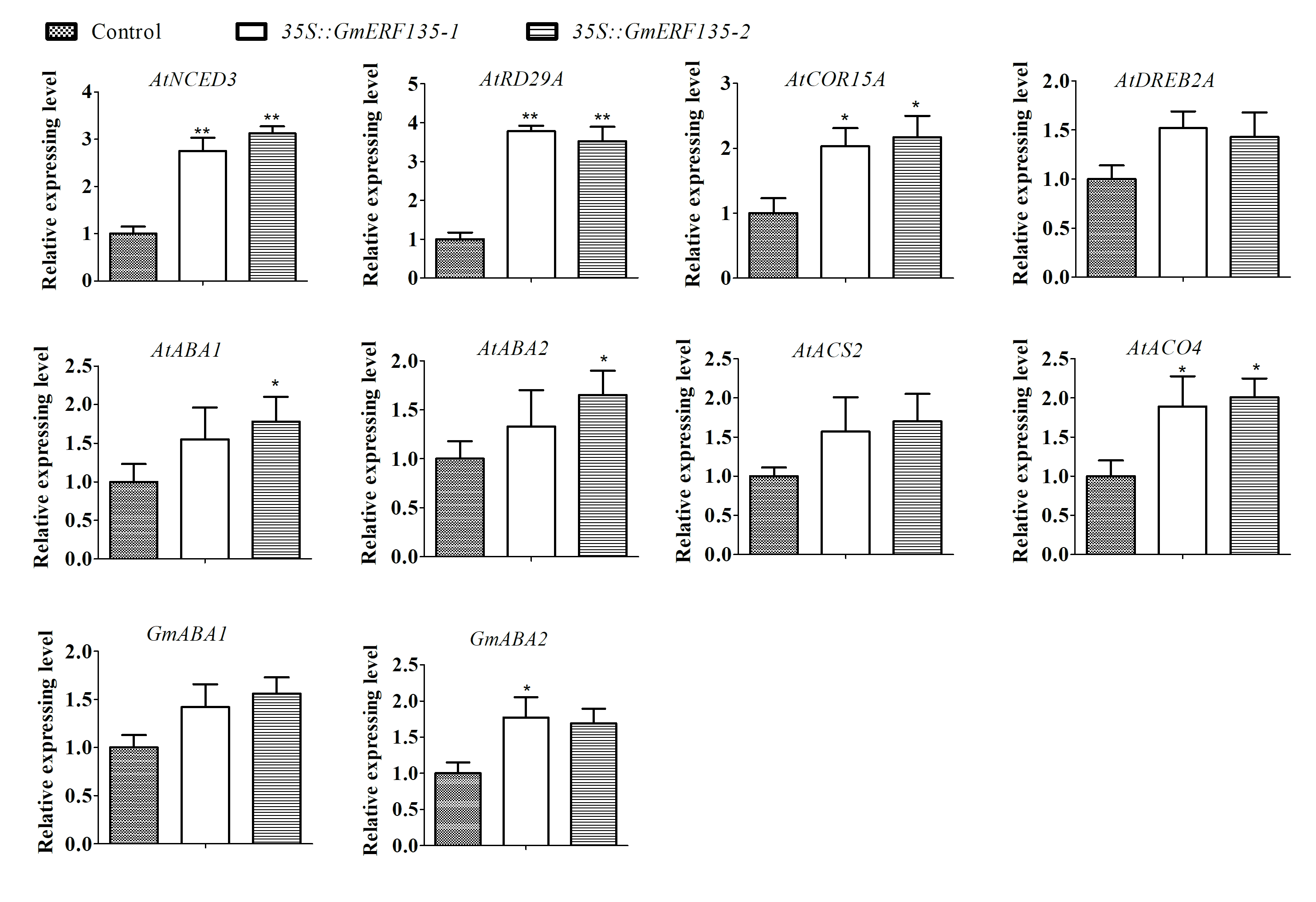

Supplement: FIGURE S2 — Express levels of various stresses-related genes after treatment. Two-week-old WT and transgenic Arabidopsis lines were used to extract total RNA. GmActin/AtActin was used for normalization. Data were shown as the means ± SDs of three experiments. [file Image_2.TIF]
